# Splinting of penis following microvascular reconstruction-A simple inexpensive method

**DOI:** 10.4103/0970-0358.59291

**Published:** 2009

**Authors:** Abhishek Sharma, Ashutosh Misra, Sandip Basu

**Affiliations:** Department of Plastic Surgery, Institute of Post Graduate Medical Education & Research, Kolkata, India

**Keywords:** Microvascular penile reconstruction, postoperative splinage

## Abstract

We present a simple method of splintage following microvascular reconstruction of penis. The splint is made by removing the bases of two thermocol glasses and joining them with paper adhesive tapes to form a hollow cylinder to protect and support the penis and keep it vertical. The splint is slid over the catheter and the reconstructed penis and fixed to the lower abdominal wall and the thighs with paper tapes for stability. A window at the base of the splint is made for the purpose of observation, while the tip is monitored from the open end at the top.

## INTRODUCTION

Difficulties in maintaining the upright position of a reconstructed penis by the microvascular technique and its monitoring have been experienced by all surgical teams performing such operations. The necessity of loose dressings combined with ability to observe the flap as and when required, demands a method which is easy to perform, does not interfere with urinary drainage, allows observation of the flap without hindrance and makes palpation and assessing temperature possible.

## MATERIAL AND METHODS

Seven patients who had microvascular reconstruction of penis done with Radial Artery Forearm flap (RAFF) between 2003 and 2007 in our institute form the material of the study. The patients were all victims of traumatic amputations of penis and reconstruction was done with modifications of radial artery forearm flap. Recipient vessels of anastomoses were deep inferior epigastric artery and vein. The second vein was either long saphenous turned over in two cases and one of its tributaries in the remaining five patients.

**Figure 1:**
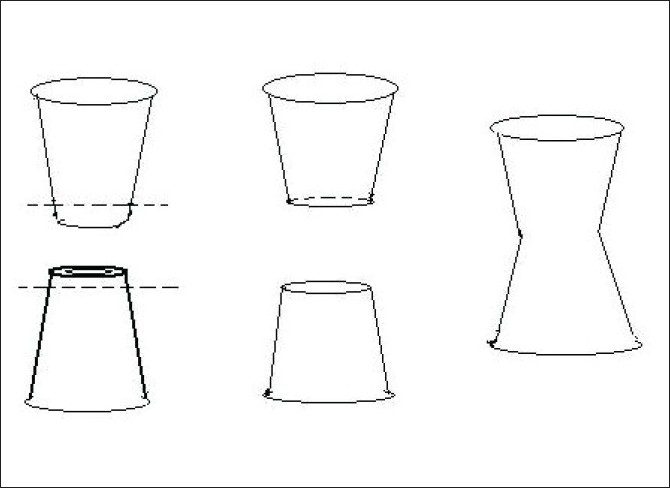
Schematic diagram of construction of splint

Two thermocol glasses were taken, their bases removed and they were attached with each other at their cut ends with the help of adhesive paper tapes (micropore). The resulting hollow cylindrical splint gave the appearance of an hourglass [Figure 1].

**Figure 2:**
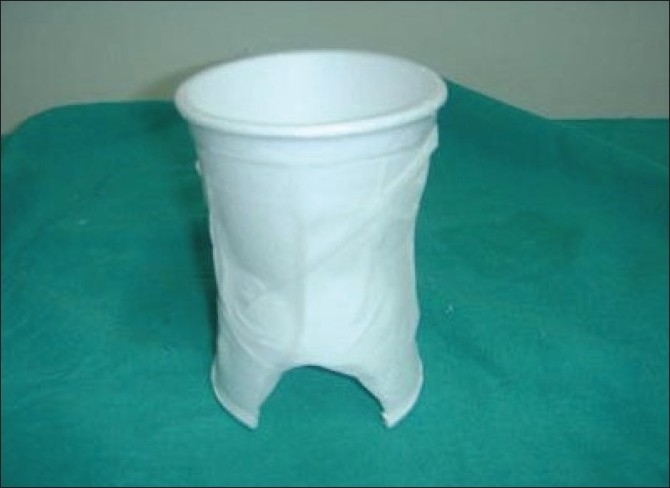
A fully constructed splint with the window at the base

**Figure 3:**
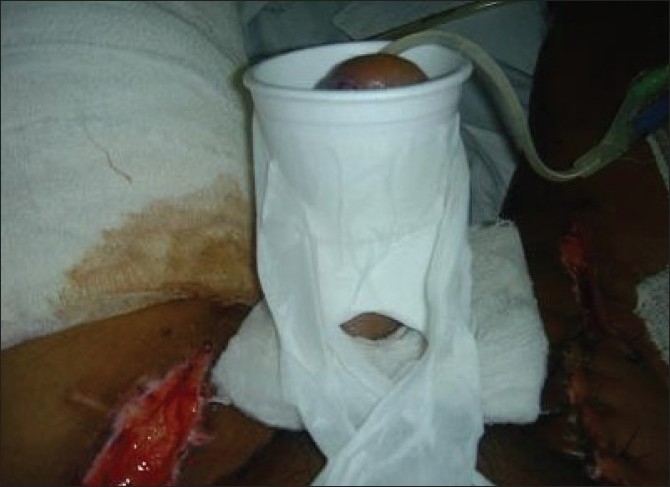
showing the visibility of the reconstructed penile base

**Figure 4:**
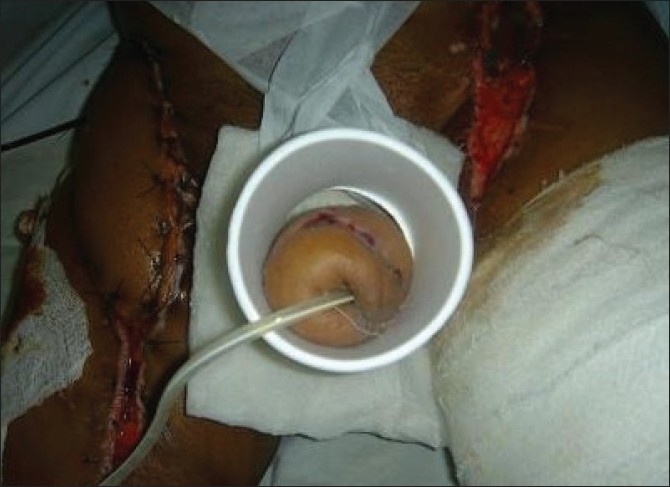
Shows ease of monitoring the flap from the top end

A small window was made in the lower part of the splint [Figure 2] to enable us to observe the flap in its lower part. After microvascular reconstruction of penis, the splint was slid over the neo-penis with a catheter and held in place by taping it to the lower abdomen [Figure 3]. The distal part of Foley's catheter was taped to the upper thigh. The space between penis and the splint was lightly packed by cotton or gauzes. The slight narrowing in the centre of the splint (waist) along with light packing around the neo-penis was helpful in keeping the penis supported and prevented it from folding on itself. This loose packing avoided any impedance to venous drainage and allowed some space for the developing oedema. The tip of the flap could easily be seen and palpated for pulsation and temperature from the top open end [Figure 4]. At the time of dressing, removal of the splint was fairly easy - by simply removing the paper tapes used to fix it with the abdomen and thigh The splint was usually kept for 2 weeks.

## RESULTS

**Figure 5:**
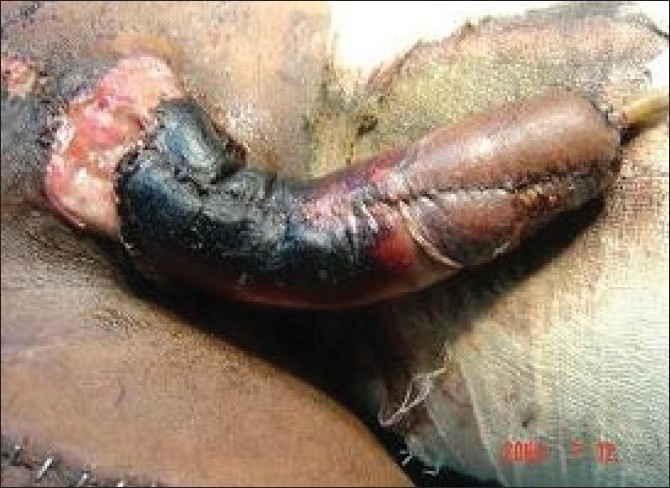
Survival of distal part, necrosis of skin in proximal part. This part could not be observed as there was no window. Subsequently the design was modified in its current form

Our experience with this thermocol-glass splint was very encouraging. As the raw materials (thermocol glasses and adhesive paper tapes) are economical and readily available, we could easily prepare this splint in the OT in a short time. The main purpose of the splintage after microvascular penile reconstruction is to keep the penis in an upright position, and monitor it from time to time. Both purposes were achieved perfectly with this splint. In the initial two cases, there was no window at the base of the splint. Unfortunately, in one of these two cases, there was skin necrosis at the base [Figure 5] resulting in urinary fistula. This prompted us to include a window in the design which we used in rest of the five cases. As the splint was light in weight, patients felt comfortable with it and also there was no incidence of displacement.

## DISCUSSION

Importance of proper splintage following microvascular penile reconstruction cannot be over emphasized. While maintenance of the penis in the upright position is necessary to facilitate venous drainage, its accidental buckling or folding onto itself may invite disaster.

Various forms of splintage and dressings following operations on penis are available including foam, POP cast, impervious materials, tie over, alginates and cut plastic syringes,[1–5] but none of them are ideal for post microsurgical reconstructions. Cut syringes have been used by Sharma *et al.* for penile resurfacing by skin grafting.[5] They used a vertically split 10 ml syringe as the splint. Tie over sutures have been placed which help to hold the penis against the splint and it gives the required compression. There are other reports of the use of disposable syringe splints[6] for skin grafting in penile reconstruction, where a 50ml syringe was slipped over the penis after cutting off its nozzle end. The skin grafted penis was covered with bolus dressings and and a 50ml syringe converted into a cylinder was slipped over this dressing to snugly fit the penis. However, in our cases after microvascular reconstruction, this snugly fitting device would have been counter productive as compression is not desired unlike in skin grafting; rather dressing should be loose enough so as to allow easy venous drainage. A plaster cylinder cast has also been suggested.[2] for immobilization of penile skin grafts but it is cumbersome while applying and taking off.

Microvascular surgeries require constant monitoring of the flap particularly observing colour and pulsation, palpating for pulsation, and temperature and sometimes pricking for observing the quality of bleeding from the flap. This thermocol splint, designed and modified to customize the penile dressing, forms an ideal splint with which all the above functions can be accomplished. The same splint can be used following any other operation on penis, whatever may be the nature of dressings. They are also economical and can be discarded and replaced at will. Thermocol is mostly inert and our splint is not in direct contact with the flap thus avoiding any form of tissue reaction to flap.

## CONCLUSION

Difficulties in maintaining the position of a reconstructed penis can be circumvented with a biconcave venturi tube type of cylindrical splint made with foam. It is light, cheap, easy to make, allows observation and easy change of dressings.
